# Immunogenicity, safety, and effectiveness of seasonal influenza vaccination in patients with diabetes mellitus: A systematic review

**DOI:** 10.1080/21645515.2018.1446719

**Published:** 2018-04-09

**Authors:** Gael Dos Santos, Halima Tahrat, Rafik Bekkat-Berkani

**Affiliations:** a GSK, Wavre, Belgium; b GSK, Rockville, MD, USA; c GSK, Philadelphia, PA, USA

**Keywords:** Diabetes, seasonal influenza, vaccination, immunogenicity, safety, vaccine effectiveness, systematic review

## Abstract

Influenza is associated with an increased risk of complications, especially in diabetic mellitus patients who are more susceptible to influenza infection. Despite recommendations of the WHO and public health authorities, vaccination uptake in this population remains suboptimal. This systematic review identified 15 studies published between January 2000-March 2017 in PubMed, Embase and Cochrane Library, which provided data on immunogenicity, safety, effectiveness, and/or cost-effectiveness of seasonal influenza vaccination in diabetic patients. Immunogenicity of seasonal influenza vaccination in diabetic patients was generally comparable to that of healthy participants. One month after vaccination of diabetic patients, seroconversion rates and seroprotection ranged from 24.0–58.0% and 29.0–99.0%, respectively. Seasonal influenza vaccination reduced the risk of hospitalization and mortality in diabetic patients, particularly those aged ≥65 years. These review results demonstrate and reinforce the need and value of annual influenza vaccination in diabetic patients, particularly in alleviating severe complications such as hospitalization or death.

## Introduction

Diabetes is a major global public health concern. Its prevalence is increasing worldwide, having more than doubled in the past 20 years.^1^ The International Diabetes Federation estimated that 1 in 11 adults or 415 million people worldwide were living with diabetes in 2015, with this figure predicted to increase to 642 million by 2040.^2^ In patients with diabetes, influenza infection is associated with an increased risk of complications, including hospitalization and death.^3-5^ Annual vaccination against seasonal influenza is therefore recommended by public health authorities worldwide for this high-risk group.^6-8^ To ensure an efficient vaccination against influenza in vulnerable populations, the World Health Organization recommends a coverage rate of at least 75% in vulnerable populations. ^9^ However, recent data suggest that seasonal influenza vaccination uptake remains suboptimal in patients with diabetes in most countries.^10-12^ One possible explanation for this finding may be lack of awareness of associated complications induced by influenza in this vulnerable group and possibly expected reduced vaccine performance due to immune dysfunction in patients with diabetes mellitus.^10^

Focusing on more severe forms of infections and associated complications, previous systematic reviews reported that all-cause hospitalization, hospitalization due to influenza or pneumonia during influenza season, and all-cause mortality were reduced in elderly diabetic patients following seasonal influenza vaccination.^13^^,^^14^ In working-age diabetic patients, vaccination was associated with reductions in all-cause hospitalization and hospitalization for influenza and pneumonia; however, no direct effects on mortality were observed. Both reviews reported that the strength of available evidence was low to very low for all outcomes and residual confounding was present in most of the included studies published until January 2015. The current systematic review was undertaken to update and expand on these previous findings with the aim to provide a critical appraisal and summary of currently available data concerning the immunogenicity, safety, efficacy, effectiveness, quality of life and cost effectiveness of seasonal influenza vaccination in patients with diabetes.

## Methods

A systematic literature review was conducted following Cochrane guidelines^15^ and Preferred Reporting Items for Systematic Reviews and Meta-Analysis (PRISMA) guidelines.^16^

### Search strategy

References for this systematic review were identified through searches in the PubMed, Embase and Cochrane Library databases from 1 January 2000 to 6 March 2017. Search strings were developed combining terms for influenza, vaccination and metabolic disorders, including diabetes mellitus. To be as extensive as possible and to collect all articles on any type of diabetes mellitus a broader search strategy on metabolic disorders was employed. In addition, various websites and data sources were searched for grey literature and the reference lists of included systematic reviews and meta-analyses were screened for additional relevant articles. The complete search strategy is shown in Appendix 1.

### Inclusion and exclusion criteria

The three databases were searched for publications on diabetic patients vaccinated against seasonal influenza. Randomized controlled trials and observational studies on the immunogenicity, safety and efficacy of seasonal influenza vaccination in patients with type 1 and type 2 diabetes of any age were included. Furthermore, prospective and retrospective observational studies on effectiveness, quality of life and/or cost effectiveness of seasonal influenza vaccination in patients with type 1 and type 2 diabetes of any age were included. No language limits were applied.

The following articles were excluded: (i) studies not describing diabetic mellitus patients or describing mixed patient groups with chronic diseases (unless the diabetic group was analyzed separately); (ii) studies in which patients received the 2009 H1N1 pandemic influenza vaccine; (iii) letters to the editor, editorials, case reports or comments; and (iv) studies of insufficient methodological quality (as outlined below).

### Study selection and critical appraisal

Articles were selected by a three-step selection procedure based on (i) screening of title and abstract, (ii) screening of full-text article, and (iii) screening during the data-extraction phase. Titles and abstracts from the database searches were independently screened by two researchers. Selected references from the two researchers were included for full text selection. The first 10% of the full text articles was critically appraised by both researchers, for the remaining articles any doubts were discussed in detail. In case of discrepancy or disagreements during the selection phase, a third researcher was consulted. The study was discussed until consensus was reached. If more than one article reported on the same study, only the most relevant or complete article was included. Both articles were included if they provided relevant complementary data. The methodological quality of included studies was critically appraised using the methodology checklists from the Scottish Intercollegiate Guidelines Network (SIGN), which are applicable for different study designs.^17^ Studies were critically appraised with a set of criteria that might have a significant effect on the risk of bias in the results reported and conclusions drawn. According to the number of risks assessed and the predicted effects on the results and conclusions, studies were classified as high, acceptable or low quality.^17^

The process of selection was registered in an Endnote library.

### Data extraction

Data from included articles were extracted into pre-defined evidence tables by one researcher and reviewed by the second researcher. Information was recorded on study characteristics (i.e. country, study design, influenza season, follow-up period and setting); study population (i.e. inclusion and exclusion criteria, gender, age and case definition); study results and critical appraisal. Depending on the objectives of the included studies, study results included data on the immune response, safety, efficacy, effectiveness, quality of life and cost effectiveness of seasonal influenza vaccination. Data from the evidence tables were used to construct summary tables per objective.

### Definitions

The immunogenicity of the influenza vaccine is most often measured in terms of hemagglutination inhibition (HI) titers, with the following parameters commonly derived in clinical trials: (1) geometric mean titer (GMT); (2) seroconversion rate (SCR); (3) seroprotection rate (SPR). SCR is usually defined as either a pre-vaccination HI titer <1:10 and a post vaccination HI titer >1:40 or a pre-vaccination HI titer ≥1:10 and a minimum four-fold rise in post-vaccination HI antibody titer. SPR is defined as the percentage of participants who attain reciprocal HI titers of ≥1:40. Predefined criteria for licensure in terms of SCR and SPR were in place at the time that most of the studies included in this review were conducted, although they are no longer considered as part of the licensure process. In the USA, the criteria for adults <65 years of age and children were that the lower limit of the two-sided 95% confidence interval (CI) of the SCR should be ≥40%, and the lower limit of the 95% CI for the SPR should be ≥70%. (CBER^18^ For adults ≥65 years of age, corresponding values were ≥30% and ≥60%. In Europe, the criteria were that the point estimates of the SCR and SPR should be ≥40% and ≥70%, respectively, in adults 18−60 years of age, and ≥30% and ≥60%, respectively, in adults >60 years of age.^19^

Vaccine efficacy is commonly defined as the direct effect of a vaccine measured in pre-licensure randomized clinical trials, where vaccination occurs under optimal and controlled conditions. It is measured as the proportionate reduction in disease attack rate among vaccinated individuals compared with unvaccinated individuals.^20^ Vaccine effectiveness is a “real world” measure of how well the seasonal influenza vaccine prevents influenza disease in the general population during a given influenza season, and is commonly assessed by observational post-licensure studies.^20^^,^^21^ Reactogenicity, which is part of the safety profile assessment of a given vaccine, refers to the potential for expected and unexpected local or systemic adverse reactions following vaccine administration.

## Results

### Study selection process

**Figure 1.**
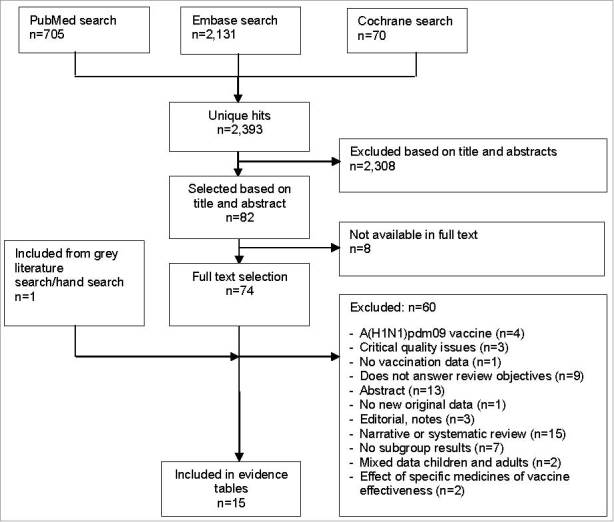
Flow chart summarizing the systematic literature search and study selection process.

The study selection process is summarized in Fig. 1. The search yielded 2,393 unique hits, from which 75 publications were considered eligible to be screened in full. In all, 15 studies met the criteria for inclusion in this systematic review.

### Study characteristics

Key characteristics of the included studies are shown in Table 1. The 15 studies included 2 randomized double-blind trials, 3 non-randomized clinical studies, 1 prospective cohort study, 7 retrospective cohort studies, and 2 case-control studies. The studies were performed over various influenza seasons during the period from 1992–2013 in Canada, Israel, Italy, Korea, the Netherlands, Spain, Taiwan, the UK and the USA. Very limited details were reported on the seasonal influenza vaccines; in seven studies a trivalent influenza vaccine was used (of which three specified the strains), in one article a subunit and virosomal vaccine were studied, and the other studies did not specify the influenza vaccine. Sample sizes ranged from 8 to 124,503 included diabetic patients. Six studies provided data on immunogenicity, two on safety, nine on vaccine effectiveness, and one on cost effectiveness. No studies providing data on vaccine efficacy or quality of life were identified. The main limitations or risks of study bias identified were the lack of descriptions of sample size calculations, significant differences in patient and clinical characteristics between study groups at baseline, potential variation in the application of diagnostic codes, the lack of influenza-specific outcomes and retrospective design. Seven studies were considered to be of acceptable quality and eight were considered to be of lower quality, according to the methodology checklists from Scottish Intercollegiate Guidelines Network (SIGN).^17^

**Table 1.** Key characteristics of included studies and results of bias risk assessment.

Reference	Country	Study design	Influenza season	Mean (SD) age^a^ in years	Gender, % male^a^	N^a^	Diabetes type	Potential bias identified & expected impact
***Immunogenicity***								
^22^	USA	Non-randomized clinical study	2011-2012	Young:^b^50 (3)/42 (2)	Young:42.9/27.0	Young: 14/37	Type 2	- Low quality
						Elderly: 8/28		+ Non-diabetic age-matched control groups
				Elderly:^b^64 (2)/66 (1)	Elderly: 50.0/35.7			- Small sample size; no sample size calculations; limited case definition; two different influenza vaccines were used
								
^23^	Canada	Non-randomized clinical study	2010-2012^c^	74.6 (6.5)/75.7 (6.6)	NR	102/119	Type 2	- Low quality
								+ Non-diabetic control group
								- Small sample size; no sample size calculations; limited information on setting and case enrolment; differences at baseline
^25^	USA	Prospective cohort study	2009-2011	≥18^d^	2009: 34.7^e^	2009: 91/461	Type 2	+ Acceptable quality
					2010: 39.3^e^	2010: 102/463		+ Non-diabetic control group
								- No sample size calculations; differences at baseline
^26^	USA	Randomized, double-blind trial	2008-2010	14.3 (6.6)	70.6	34	Type 1	- Low quality
								- Small sample size, no sample size calculations, limited information on setting and enrolment
								- Primary study objective was to assess the effect of abatacept on immunogenicity of vaccines in patients with type 1 diabetes; no non-diabetic control group
***Immunogenicity and safety***								
^24^	Korea	Non-randomized clinical study	2012–2013	63.0 (9.7)/60.0 (14.1)	48.6/29.2	105/108	Type 2	- Low quality
								+ Non-diabetic control group
								- Small sample size; limited case definition; differences at baseline
^27^	Italy		Randomized, double-blind trial	2007-2008	15.8 (5.3)/16.2 (3.5)^f^			49.1/51.9^f^	53/52^f^	Type 1	+ Acceptable quality
								- Small sample size; no sample size calculations			
								- Comparative study of two different seasonal influenza vaccines; no non-diabetic control group			
***Effectiveness***											
^36^	UK	Retrospective cohort study	2003–2010	66.2 (13.3)/56.2 (16.3)^g^	53.9/54.2^g^	124,503^h^	Type 2	+ Acceptable quality			
								+ Unvaccinated diabetic control group			
								- No sample size calculations; differences at baseline; diagnostics code; no information on influenza seasons			
^33^	Spain	Retrospective cohort study	2002-2005	75.2 (6.5)/73.1 (6.9)	42.2/39.8	1,586/1,064	Type 1 & 2^i^	- Low quality			
								+ Unvaccinated diabetic control group			
								- No sample size calculations; differences at baseline; no description of diabetes types; incomplete data; diagnostic codes; no influenza-specific outcomes			
^30^	Israel	Retrospective cohort study	2000-2001	72.8 (0.6)/73.1 (0.5)^j^	51.8/42.1^j^ 46.6/40.4 ^j^	15,556/69,097	Type 1 & 2^i^	- Low quality			
				74.7 (0.8)/74.2 (0.5)^j^							
								- No sample size calculations; retrospective design; no description of diabetes types; diagnostic codes; limited information on exposure assessment; univariate analysis; no influenza-specific outcomes; no information on influenza season			
^32^	The Netherlands	Nested case-control study	1999-2000	68.1 (13.7)/69.8 (12.6)^k^	51.6/38.3^k^	192/1,561^k^	Type 1 & 2 (>90% Type 2)	+ Acceptable quality			
								- No sample size calculations; differences at baseline; diagnostic codes; no influenza-specific outcomes			
^31^	Canada	Retrospective cohort study	2000-2008	53/74/74^l,^^m^	52/47/44^m^	70,380/50,308/76,233^m^^,^^n^	Type 1 & 2^i^	+ Acceptable quality			
								+ Unvaccinated diabetic control group			
								- No sample size calculations; retrospective design; no description of diabetes types; diagnostic codes; no information on influenza seasons			
^28^	USA		Retrospective cohort study	1996-1998	≥65^d^		NR	1996: 14,915	Type 1 & 2^i^	+ Acceptable quality	
						1997: 21,991		- Differences at baseline; retrospective design; no description of diabetes types; diagnostic codes			
^29^	USA		Case-control study	1992-1998	72.9/73.7^l,^^o^		32.2/34.7^o^	1,735/180^o^	Type 1 & 2^l^	- Low quality	
								- No sample size calculations; differences at baseline; no description of diabetes types; diagnostic codes; no influenza-specific outcomes; no information on influenza seasons			
^34^	The Netherlands		Retrospective cohort study	1999-2000	58.6^q^		1999: 49	1999: 2,944	Type 1 & 2^i^	- Low quality	
				2001–2002^p^			2001: 50	2001: 5,035		- No sample size calculations; retrospective design; no description of diabetes types; diagnostic codes; no influenza-specific outcomes; no information on matching	
*Effectiveness and cost-effectiveness*											
^35^	Taiwan		Retrospective cohort study	2001-2009	73.1 (5.9)/73.2 (6.8)	50.0/49.5	4,571/4,454	Type 1 & 2^i^	+ Acceptable quality		
								+ Unvaccinated diabetic control group			
								- No sample size calculations; retrospective design; differences at baseline; no description of diabetes types; diagnostic codes; no information on influenza seasons			

n: number of participants; NR: not reported; RCT: randomized controlled trial; SD: standard deviation; UK: United Kingdom; USA: United States of America

a Age and gender presented as vaccinated/unvaccinated or diabetics/non-diabetics where available;

b Young (20–59 years), Elderly (>60 years);

c Only one season per subject;

d Mean age not reported;

e For the total study population;

f *Subunit/virosomal vaccine*;

g In season 2003/2004;

h Total number of patients enrolled, including a total of 623,591 Person-Years;

i No data on the proportion of patients with type 1 or type 2 diabetes;

j Vaccinated vs unvaccinated patients with diabetes and vaccinated vs unvaccinated in subjects without diabetes;

k Case participants vs controls;

^l^ Median age;

m Working-age diabetic participants, elderly diabetic participants and elderly without diabetes;

n Included person-years; Data were collected annually;

o Controls vs myocardial infarction cases;

p In 2000–2001, no epidemic occurred and therefore no data were collected;

q In high risk groups, no mean age of patients with diabetes available.

### Immunogenicity

Six studies provided data on the immunogenicity of seasonal influenza vaccination in diabetic patients (one randomized double-blind trial of acceptable quality, one randomized double-blind trial of low quality, three non-randomized clinical studies of low quality and one prospective cohort study of acceptable quality).^22-27^ Four studies included a control group of healthy participants.^22-25^ Two of these studies assessed immunogenicity by change in HI titers four weeks after vaccination; both found the immune response of diabetic patients to be comparable to that of healthy participants.^22^^,^^23^ Immunogenicity was not found to decrease with age in patients with diabetes, when comparing results of adult patients (mean age of 50 years) with results of elderly patients (mean age of 64 years).^22^ In a population of elderly patients (≥65 years old) good glycemic control was shown to be important to accurately measure immune response to seasonal influenza vaccination in this population.^23^ However, the studies of Frasca et al. and McElhaney et al. might not be comparable in key characteristics of the study populations, such as control of the disease.

The four other studies presented SCR and/or SPR one month after vaccination (Table 2 and Figure 1S).^24-27^ SCR ranged from 44.0–54.9% for the A/H1N1 strain, 27.7–58.0% for the A/H3N2 strain and 24.0–54.3% for the B strain. SCR were above the criterion for licensure (i.e. >40%) for all vaccine strains in all studies, except for the A/H3N2 strain in one study^27^ and for the B strain during a second influenza season in another study.^25^ No significant differences in SCR at one-month post-vaccination for individual vaccine strains were seen between patients with diabetes and healthy participants in studies which included a control group, except against the B strain in one study (24.0% versus 31.9%, respectively; p = 0.05).^25^ Two studies also measured SCR six months after vaccination.^24^^,^^27^ At this timepoint, SCR for most vaccine strains were below the recommended thresholds (Table 2). No significant differences in SCR at this timepoint were reported between patients with diabetes and healthy participants in the study which included a control group.^24^

**Table 2.** Seroconversion and seroprotection rates one and six months after seasonal influenza vaccination in patients with diabetes.

Reference	Country	Study design	Influenza season	Vaccine type	Influenza type/subtype	n	Patient characteristics	Immunogenicity outcome % (n/N or 95% CI)
**Seroconversion rate (1 month)**								
^24^ (SIGN -)	Korea	Non-randomized clinical study	2012-2013	TIV	A/H1N1	105	Type 2 / ≥19 years	54.3% (57/105)
					A/H3N2	105		46.7% (49/105)
					B	105		54.3% (57/105)
^25^ (SIGN +)	USA	Prospective cohort study	2009-2010	TIV			Type 2 / ≥18 years	*Year 1*
					A/H1N1	91		54.9% (NR)
					A/H3N2	91		57.7% (NR)
					B	91		40.0% (NR)
			2010–2011					*Year 2*
					A/H1N1	102		44.0% (NR)
					A/H3N2	102		58.0% (NR)
					B	102		24.0% (NR)^*^
^27^ (SIGN +)	Italy	Randomized, double-blind trial	2007-2008	Subunit	A/H1N1	53	Type 1 / 9–30 years	44.7% (30.2–59.9)
					A/H3N2	53		27.7% (15.6–42.6)^*^
					B	53		42.6% (28.3–57.8)
				Virosomal				
					A/H1N1	52		50.0% (35.2–64.8)
					A/H3N2	52		31.2% (18.7–46.2)^*^
					B	52		50.0% (35.2–64.8)
**Seroconversion rate (6 months)**								
^24^ (SIGN −)	Korea	Non-randomized clinical study	2012–2013	TIV	A/H1N1	105	Type 2 / ≥19 years	26.7% (28/105)^*^
					A/H3N2	105		34.3% (36/105)^*^
					B	105		32.4% (34/105)^*^
^27^ (SIGN +)	Italy	Randomized, double-blind trial	2007–2008	Subunit	A/H1N1	53	Type 1 / 9–30 years	42.6% (28.3–57.8)
					A/H3N2	53		14.9% (6.2–28.3)^*^
					B	53		25.5% (13.9–40.3)^*^
				Virosomal	A/H1N1	52		41.7% (27.6–56.8)
					A/H3N2	52		20.8% (10.5–35.0)^*^
					B	52		45.8% (31.4–60.8)
**Seroprotection rate (1 month)**								
^24^ (SIGN -)	Korea	Non-randomized clinical study	2012-2013	TIV	A/H1N1	105	Type 2 / ≥19 years	69.5% (73/105)^*^
					A/H3N2	105		99.0% (104/105)
					B	105		56.2% (59/105)^*^
^25^ (SIGN +)	USA	Prospective cohort study	2009-2010	TIV			Type 2 / ≥65 years	*Year 1*
					A/H1N1	91		76.1% (NR)
					A/H3N2	91		85.9% (NR)
					B	91		42.3% (NR)^*^
			2010–2011	TIV			Type 2 / ≥65 years	*Year 2*
					A/H1N1	102		46.0% (NR)^*^
					A/H3N2	102		77.0% (NR)
					B	102		29.0% (NR)^*^
^26^ (SIGN -)	USA	Randomized, double-blind trial	2008-2010	TIV			Type 1 / 7–35 years	*Year 1*
					A/H1N1	29		89.7% (NR)
					A/H3N2	29		93.1% (NR)
					B	29		72.4% (NR)
								*Year 2*
					A/H1N1	16		87.5% (NR)
					A/H3N2	16		93.8% (NR)
					B	16		43.8%^*^ (NR)
^27^ (SIGN +)	Italy	Randomized, double-blind trial	2007-2008	Subunit	A/H1N1	53	Type 1 / 9–30 years	100% (92.4-100)
					A/H3N2	53		95.7% (85.5-99.5)
					B	53		70.2% (55.1-82.7)
				Virosomal	A/H1N1	52		100% (92.6-100)
					A/H3N2	52		100% (92.6-100)
					B	52		72.9% (58.1-84.7)
**Seroprotection rate (6 months)**								
^24^ (SIGN -)	Korea	Non-randomized clinical study	2012-2013	TIV	A/H1N1	105	Type 2 / ≥19 years	43.8% (46/105)^*^
					A/H3N2	105		95.2% (100/105)
					B	105		31.4% (33/105)^*^
^27^ (SIGN +)	Italy	Randomized, double-blind trial	2007-2008	Subunit	A/H1N1	53	Type 1 / 9–30 years	93.6% (82.5–98.7)
					A/H3N2	53		95.7% (85.5–99.5)
					B	53		46.8% (32.1–61.9)^*^
				Virosomal	A/H1N1	52		91.7% (80.0–97.7)
					A/H3N2	52		95.8% (85.7–99.5)
					B	52		62.5% (47.3–76.0)^*^

CI: confidence interval; N: number; n/N: subgroup/total study population; NR: not reported; TIV: trivalent inactivated vaccine; USA: United States of America.

* According to immunogenicity criteria for licensure: seroconversion rate should be >40% and seroprotection rate should be >70% in adults 18–60 years, seroconversion rate should be >30% and seroprotection rate should be >60% in adults >60 years.

SPR one month after vaccination ranged from 46.0–100% for the A/H1N1 strain, 77.0–100% for the A/H3N2 strain and 29.0–72.9% for the B strain (see Table 2 and Figure 2S).^24-27^ SPR for the A/H1N1 and A/H3N2 strains were generally above the criterion for licensure (i.e. >70%); however, SPR for the B strain tended to be lower. No significant differences in SPR for individual vaccine strains were seen between patients with diabetes and healthy participants in studies which included a control group, except for the B strain during a second influenza season in one study (29.0% versus 40.7%, respectively; p = 0.04).^25^ SPR six months after seasonal influenza vaccination generally remained high for the A/H1N1 and A/H3N2 strains, but decreased compared with the rates initially observed one month after vaccination.^24^^,^^27^ No significant differences in SPR for the A/H3N2 and B strains at this timepoint were seen between patients with diabetes and healthy participants in the study which included a control group; however, the SPR for the A/H1N1 strain was significantly lower in patients with diabetes (43.8% versus 59.3% in healthy participants; p = 0.028).^24^

### Effectiveness

Nine studies (two case-control studies [one of acceptable quality and one of low quality] and seven retrospective cohort studies [four of acceptable quality and three of low quality]) provided data on seasonal influenza vaccine effectiveness in patients with diabetes.^28-36^

Five studies reported the effect of seasonal influenza vaccination on all-cause mortality in patients with diabetes (Table 3).^30^^,^^32^^,^^33^^,^^35^^,^^36^ All found seasonal influenza vaccination to be effective in preventing mortality in diabetic patients, particularly those aged ≥65 years. Vamos et al.^36^ found that seasonal influenza vaccination was associated with a 50% reduction in all-cause mortality during influenza seasons over the 7-year study period in adult patients with type 2 diabetes (incidence rate ratio [IRR], 0.50 [95% CI 0.45-0.54]; *p* ≤ 0.001). Excluding the 2008/09 cohort (preceding the pandemic when the A/H1N1_pdm09_ influenza strain was circulating) from the analysis did not impact the IRR obtained. In the study by Wang et al.,^35^ the hazard ratio for all-cause mortality was 0.40 (95% CI 0.29-0.56) in patients aged 65–74 years and 0.46 (95% CI 0.35-0.59) in those aged ≥75 years who received influenza vaccination as compared to non-vaccinated. In the study reported by Rodriguez-Blanco et al.,^33^ a multivariate adjusted analysis combining results of four influenza seasons (2002-2005) produced an odds ratio (OR) for all-cause mortality of 0.67 (95% CI 0.47-0.96) in diabetic patients aged ≥65 years who received influenza vaccination. Heymann et al.^30^ reported that the ORs of death for elderly male and female patients were 0.35 (95% CI 0.25-0.49) and 0.32 (95% CI 0.20-0.50), respectively. Looijman-Van den Akker et al.^32^ reported that vaccine effectiveness for the prevention of all-cause mortality during influenza season was 56% (95% CI 4%-80%) in diabetic patients aged ≥65 years compared with 24% (95% CI -706%-93%) in those aged 18–64 years.

**Table 3.** Effectiveness of seasonal influenza vaccination for the prevention of mortality and hospitalization in patients with diabetes.

Reference	Country	Study design	Influenza season	n	Diabetes type	Subgroup analysis	Effectiveness outcome
							Mortality
**^36^** (SIGN +)	UK	Retrospective cohort study	2003-2010	107,598.2 PY	Type 2		*IRR (95% CI) all-cause mortality*
							0.50 (0.45-0.54) [0.52 (0.47-0.58)]^a^
**^35^** (SIGN +)	Taiwan	Retrospective cohort study	2001-2009		Type 1 & 2		*IRR (95% CI) all-cause mortality*
				5,954		65–74 years	0.40 (0.34-0.47)
				3,071		≥75 years	0.41 (0.34-0.50)
							*HR (95% CI) all-cause mortality*
				5,954		65–74 years	0.40 (0.29-0.56)
				3,071		≥75 years	0.46 (0.35-0.59)
**^33^** (SIGN -)	Spain	Retrospective cohort study	2002	1,586	Type 1 & 2	≥65 years	*Incidence (95% CI) all-cause mortality per 1,000 person-influenza period*
				1,064		Vaccinated	7.6 (3.9-13.2)
						Unvaccinated	11.3 (5.9-19.7)
			2003	1,682		Vaccinated	16.7 (11.2-24.2)
				876		Unvaccinated	21.7 (13.3-34.1)
			2004	1,715		Vaccinated	14.6 (9.5-21.6)
				707		Unvaccinated	18.4 (9.9-31.5)
			2005	1,629		Vaccinated	25.2 (18.2-34.8)
				657		Unvaccinated	25.9 (15.1-42.1)
						Overall	*RRR (95% CI) all-cause mortality*
			2002	2,650			0.67 (0.29-1.48)
			2003	2,558			0.77 (0.43-1.39)
			2004	2,422			0.79 (0.41-1.56)
			2005	2,286			0.97 (0.48-1.82)
			2002–2005	9,916		Overall	*Multivariate adjusted OR (95% CI) all-cause mortality*
							0.67 (0.47–0.96)
**^30^** (SIGN -)	Israel	Retrospective cohort study	2000-2001		Type 1 & 2	*Men*	*OR (95% CI) all-cause mortality*
				5,195		65–75 years	0.37 (0.21–0.66)
				2,278		>75-85 years	0.36 (0.22–0.58)
				456		>85 years	0.28 (0.12–0.64)
				7,929		Overall	0.35 (0.25–0.49)
						*Women*	*OR (95% CI) all-cause mortality*
				5,215		65–75 years	0.39 (0.21–0.73)
				2,713		>75-85 years	0.09 (0.03-0.24)
				526		>85 years	0.57 (0.24–1.33)
				8,454		Overall	0.32 (0.20–0.50)
**^32^** (SIGN +)	The Netherlands	Nested case-control study	1999-2000	439	>90% Type 2	*18-64 years*	*IR all-cause mortality*
						Vaccinated	2.0
						Unvaccinated	3.1
				1,314		*≥65 years*	*IR all-cause mortality*
						Vaccinated	8.4
						Unvaccinated	18.7
				1,753		≥18 years	*Adjusted VE (95% CI)*
							58% (13-80)
				439		18–64 years	24% (-706-93)
				1,314		≥65 years	56% (4-80)
							Hospitalisation
**^36^** (SIGN +)	UK	Retrospective cohort study	2003-2010	108,231.6 PY	Type 2		*IRR (95% CI) hospitalization for myocardial infarction*
							0.78 (0.65-0.93) [0.76 (0.62-0.93)]^a^
				108,282.8 PY			*IRR (95% CI) hospitalization for stroke*
							0.82 (0.67-1.00) [0.86 (0.69-1.07)]
				107,700.7 PY			*IRR (95% CI) hospitalization for heart failure*
							0.83 (0.74-0.93) [0.82 (0.72-0.93)]
				107,657.9 PY			*IRR (95% CI) hospitalization for pneumonia/influenza*
							0.75 (0.68-0.82) [0.76 (0.68-0.85)]
**^35^** (SIGN +)	Taiwan	Retrospective cohort study	2001-2009		Type 1 & 2		*IRR (95% CI) any hospitalization*
				5,954		65–74 years	0.96 (0.86-1.09)
				3,071		≥75 years	0.84 (0.72-0.98)
							*IRR (95% CI) hospitalization for pneumonia/influenza*
						65–74 years	0.98 (0.86-1.12)
						≥75 years	0.77 (0.65-0.92)
							*IRR (95% CI) hospitalization for respiratory failure*
						65–74 years	0.98 (0.83-1.16)
						≥75 years	0.50 (0.41-0.62)
							*IRR (95% CI) intensive care unit*
						65–74 years	0.37 (0.30-0.45)
						≥75 years	0.25 (0.19-0.33)
							*HR (95% CI) any hospitalization*
				5,954		65–74 years	0.91 (0.81-1.02)
				3,071		≥75 years	0.85 (0.75-0.96)
							*HR (95% CI) hospitalization for pneumonia/influenza*
						65–74 years	0.93 (0.80-1.09)
						≥75 years	0.78 (0.65-0.93)
							*HR (95% CI) hospitalization for respiratory failure*
						65–74 years	0.97 (0.63-1.49)
						≥75 years	0.55 (0.40-0.76)
							*HR (95% CI) intensive care unit*
						65–74 years	0.40 (0.29-0.56)
						≥75 years	0.46 (0.35-0.59)
**^30^** (SIGN -)	Israel	Retrospective cohort study	2000-2001		Type 1 & 2	*Men*	*OR (95% CI) hospitalization*
						65–75 years	0.99 (0.81-1.22)
						75–85 years	0.80 (0.62-1.05)
						>85 years	0.48 (0.25-0.95)
						*Women*	*OR (95% CI) hospitalizations*
						65–75 years	0.85 (0.68-1.04)
						75–85 years	0.79 (0.61-1.02)
						>85 years	0.92 (0.51-1.66)
**^32^** (SIGN +)	The Netherlands	Nested case-control study	1999-2000	439	>90% Type 2	*18-64 years*	*IR hospitalization*
						Vaccinated	12.0
						Unvaccinated	25.2
				1,314		*≥65 years*	*IR hospitalization*
						Vaccinated	13.9
						Unvaccinated	11.2
							*Adjusted VE (95% CI) against hospitalization*
				1,753		≥18 years	54% (26-71)
				439		18–64 years	70% (39-85)
				1,314		≥65 years	14% (-88-60)
**^31^** (SIGN +)	Canada	Retrospective cohort study	2000-2008		Type 1 & 2		*IRR (95% CI) all hospitalizations*
				70380 PY		18–64 years	0.72 (0.68-0.76)
				50308 PY		≥65 years	0.67 (0.64-0.70)
							*IRR (95% CI) hospitalization for pneumonia*
				70380 PY		18–64 years	0.57 (0.46-0.72)
				50308 PY		≥65 years	0.55 (0.47-0.66)
							*IRR (95% CI) hospitalization for influenza-like illness*
				70380 PY		18–64 years	0.99 (0.97-1.01)
				50308 PY		≥75 years	0.87 (0.84-0.90)

CI: confidence interval; HR, hazard ratio; IR, incidence rate; IRR, incidence rate ratio; n: number; OR, odds ratio; PY, person-years; RRR, relative risk ratio; UK: United Kingdom; VE: vaccine effectiveness.

a Excluding 2008/2009 when the outbreak of pandemic A/H1N1_pdm09_ occurred;

b Hospitalizations for physician-diagnosed influenza, pneumonia, other acute respiratory disease, myocardial infarction, heart failure, stroke or diabetes dysregulation.

Five studies provided data on the effect of seasonal influenza vaccination on the risk of hospitalization in patients with diabetes mellitus (Table 3).^30^^,^^31^^,^^32^^,^^35^^,^^36^ Seasonal influenza vaccination was associated with significantly lower admission rates for acute myocardial infarction (MI) (IRR, 0.78 [95% CI 0.65-0.93]; *p* ≤ 0.01), heart failure (IRR, 0.83 [95% CI 0.74-0.93]; *p* ≤ 0.001) and pneumonia/influenza (IRR, 0.75 [95% CI 0.68-0.82]; *p* ≤ 0.001) and a reduction (although not statistically significant) in hospital admissions for stroke (IRR, 0.82 [95% CI 0.67-1.00]), during influenza seasons over a 7-year study period in adult patients with type 2 diabetes.^36^ Seasonal influenza vaccination was effective for the prevention of hospitalization for pneumonia or influenza in diabetic patients aged 18–64 years, ≥65 years and ≥75 years in two studies,^31^^,^^35^ as well as for the prevention of hospitalization for respiratory failure in diabetic patients aged ≥75 years and intensive care unit admission in those aged ≥65 years in one of these studies.^35^ In another study, seasonal influenza vaccine was effective for the prevention of hospitalization in diabetic patients aged 18–64 years, but not in those aged ≥65 years. (Looijmans-Van et al.^32^ Vaccine effectiveness for the prevention of hospitalizations due to influenza, pneumonia, other acute respiratory disease, MI, congestive heart failure, stroke or diabetes was 70% (95% CI 39–85%) in patients aged 18–64 years compared to 14% (95% CI -88-60%) in those aged ≥65 years. This difference might be explained by differences in key characteristics of the younger and older patients. Elderly patients experience senescence or biological ageing (i.e. which refers to the gradual decline of functions’ characteristics linked specifically to cells or to the whole organism).^32^ In another study, deemed low quality, seasonal influenza vaccination was only effective for the prevention of hospitalization in internal medicine and geriatric wards for male patients aged >85 years, but not in male patients in other age groups or females.^30^

In another study, seasonal influenza vaccination was found to be effective in reducing the risk of hospitalization for pneumonia or influenza and mortality from all causes in elderly patients with diabetes over two influenza seasons.^28^ Vaccine effectiveness was 50% (95% CI 37–60%) in the first year and 21% (95% CI 6–34%) in the second year in diabetic patients compared with 46% (95% CI 34–56%) and 42% (95% CI 25–52%) in the two years, respectively, in healthy elderly participants.

Three studies provided data on effectiveness outcomes other than mortality or hospitalization.^29^^,^^31^^,^^34^ In one study, a significant association was found between seasonal influenza vaccination and the incidence of influenza-like illness in diabetic patients aged ≥65 years, but no association was found in patients aged 18–64 years.^31^ In a second study conducted over two influenza seasons, no difference in the number of general practitioner (GP) visits was found between vaccinated and unvaccinated patients with diabetes during a mild season; however, in a more virulent influenza season, vaccinated patients visited the GP less often than unvaccinated patients.^34^ In the third study, the association between influenza vaccination and risk of incident MI in persons at least 65 years of age with and without diabetes was analyzed. This study does not provide evidence supporting an association of influenza vaccination and reduction in risk of MI.^29^

### Cost effectiveness

One study of acceptable quality reported that seasonal influenza vaccination reduced the cost of hospitalization in patients with diabetes by US$1283 (95% CI -2210, -355) compared with unvaccinated diabetic patients.^35^

### Safety

Two studies provided data on the reactogenicity and/or safety of seasonal influenza vaccination in diabetic patients.^24^^,^^27^ In a randomized controlled trial of acceptable quality which compared the reactogenicity of a trivalent inactivated vaccine with a virosomal vaccine in children with type 1 diabetes, only transient and non-severe adverse reactions were reported in both groups.^27^ A non-randomized clinical study of low quality compared adverse events following seasonal influenza vaccination in patients with type 2 diabetes with those in healthy participants.^24^ All local reactions (i.e. injection-site pain, tenderness, redness and swelling) were less frequently reported by diabetic individuals than by nondiabetics, although systemic reactions (i.e. headache, malaise, chills, tiredness, sweating, myalgia and arthralgia) were reported at similar rates in the two groups.

## Discussion

This comprehensive systematic literature review was undertaken to critically assess currently available data on the immunogenicity, safety, efficacy, effectiveness, quality of life and cost effectiveness of seasonal influenza vaccination in diabetic patients. Although some evidence is missing and limited for some endpoints, the overall findings consistently support the benefit of seasonal influenza vaccination in this population. In addition, in most of the identified studies, the immunogenicity of seasonal influenza vaccination in diabetic patients was found to be comparable to that in healthy participants or non-diabetic participants.^22-27^ SCR and SPR one month after vaccination were generally above the recommended criteria for licensure (>40% and >70, respectively), with however some variation in SCR and SPR seen across studies most likely reflecting differences in the degree of similarity between the vaccine strains and predominant circulating strains during study periods. In terms of safety, seasonal influenza vaccination was found to be well-tolerated in both children with type 1 diabetes and in adults with type 2 diabetes,^24^^,^^27^ which is aligned with the known safety profile of seasonal influenza vaccines in healthy adults.^37^ During the selection phase, the study of^38^ was identified, it did not fit the inclusion criteria.^38^ This small study compared the antibody response to A/H3N2 for adults with type 2 diabetes versus non-diabetic subjects and did not find differences between these two populations in the immune response measured.

Seasonal influenza vaccination was found to be associated with a significantly reduced risk of all-cause mortality in diabetic patients, particularly older adults aged ≥65 years. Previous systematic reviews highlighted lack of adjustment for residual confounding as a major limitation of most studies of the effectiveness of influenza vaccination in diabetic patients.^13^^,^^14^

In the largest study included in this systematic review, published more recently — a retrospective cohort study involving 125,503 adults with type 2 diabetes — all-cause mortality was 24% lower during the influenza season in vaccine recipients as compared with unvaccinated participants after adjustment for residual confounding (IRR 0.76, 95% CI 0.65-0.83).^36^

Seasonal influenza vaccination was also found to be associated with significant reductions in the risk of hospitalization in diabetic patients in most studies included in the current systematic review. After adjustment for residual confounding in the large retrospective cohort study of adult patients with type 2 diabetes (n = 124,503), vaccination was associated with a 22% reduction during the influenza season in the rate of hospital admissions for acute MI (IRR 0.78, 95% CI 0.65-0.93), 18% for stroke (IRR 0.82, 95% CI 0.67-1.00), 17% for heart failure (IRR 0.83, 95% 0.74-0.93) and 25% for pneumonia or influenza (IRR 0.75, 95% CI 0.68-0.82).^36^ Cost-effectiveness data were sparse; however, one study conducted on universal claims data from 2001 and 2009 and encompassing almost 9,000 diabetic patients found hospitalization costs to be significantly lower in vaccinated diabetic patients than in unvaccinated patients with diabetes.^35^

A major strength of this systematic review is that it provides data on a wider range of clinically relevant outcomes than previous analyses.^13^^,^^14^ This literature review attempts to present and appraise several components including clinical trials, observational studies and cost-effectiveness studies. For example, immunogenicity data were also included in the present review to give a more complete overview of the available data on seasonal influenza vaccination in patients with diabetes. This is an important addition because healthcare professionals might be reluctant to vaccinate diabetic patients or patients themselves to receive the vaccination if they are not fully aware of the associated risks in case of infections or do not fully know the benefit of seasonal vaccination. An additional strength is the inclusion of studies which only considered a population of diabetic patients (or separate analysis of the diabetic patient group) allowing for a targeted assessment of the available evidence; this is important as patients with different medical conditions might vary in their response to the vaccine.

This review encompasses some limitations. The paucity of data available for some outcomes; such as the safety or the cost-effectiveness component which limits the possibility to have an overview of the safety profiles or to consistently measure the financial impact of the vaccination in this risk group; only two studies reported safety data^24^^,^^27^ and a single study provided cost-effectiveness data.^35^ No studies providing data on vaccine efficacy or quality of life were identified. Furthermore, the included studies were heterogeneous in terms of the considered populations (age groups, diabetes type, healthcare setting), settings, influenza seasons covered, outcomes and the outcome measures used, making it difficult to compare findings between studies. Another limitation is the fact that the included studies were conducted during different influenza seasons from 1992–2013. Influenza seasons vary in length and severity, which may lead to differences in findings unrelated to the population studied. Every season, the influenza vaccine is potentially re-formulated based on WHO recommendations as influenza viruses evolve continuously. The degree to which the strains included in the vaccine match the circulating strains has a large impact on the effectiveness of the vaccine during a season.^39^ In this review, it was not possible to control for this by segregating results according to the degree of matching between strains included in the vaccine and circulating strains due to the relatively low number of included studies. On the other hand, this work provides a picture of more than 10 years of a consistent trend supporting the value of seasonal influenza vaccination in patients with diabetes mellitus. In addition, no limits were set on sample size during study selection, meaning some relatively underpowered studies were included. The smallest was a non-randomized clinical study in which data were stratified on age and the sample of elderly diabetic patients involved eight participants,^22^ while the largest was a retrospective 7-year cohort study of 124,503 adults with type 2 diabetes.^36^

No high-quality randomized controlled trials of seasonal influenza vaccination in diabetic patients were identified for inclusion in this review. Of the included studies, seven were considered to be of acceptable quality and eight were considered to be of lower quality. Potential sources of bias were identified in many studies, including lack of sample size calculations, significant differences in patient and clinical characteristics between study groups at baseline, variability in application of diagnostic codes, lack of influenza-specific outcomes and retrospective design. Selection bias may be a limitation of the included observational studies, which might result in large differences in baseline characteristics between vaccinated and unvaccinated patients. For example, vaccine uptake may be higher in patients with more severe diseases as they might be more likely to recognize the value of vaccination and their doctors might be more incline to encourage influenza vaccination. In contrast, in vaccine studies the healthy vaccinee effect cannot be excluded, assuming that a patient who adopts healthy behaviors may be more likely to receive the vaccine and/or might be more prone to also seek other preventive measures and therefore may have better outcomes than unvaccinated and less health-conscious counterparts^40^; this effect being more likely to occur in the broad population, rather than in a vulnerable group recommended for annual vaccination. Measures for accounting for potential differences at baseline are crucial during statistical analysis; nevertheless, residual confounding cannot be completely ruled out in observational studies.

In conclusion, this systematic literature review brought additional inputs and provides significant evidence of the value of seasonal influenza vaccination in diabetic patients, particularly for the prevention of severe disease complications. These findings consistently support existing recommendations for seasonal influenza vaccination in this vulnerable patient population.

## Supplementary Material

KHVI_A_1446719_Supplemental.docx
