# Supplementary material for: Immunogenicity, safety, and effectiveness of seasonal influenza vaccination in patients with diabetes mellitus: A systematic review
Source: Hum Vaccin Immunother. 2018 Apr 9;14(8):1853–66. doi: 10.1080/21645515.2018.1446719 (PMC6149986; doi:10.1080/21645515.2018.1446719)
Supplement: KHVI_A_1446719_Supplemental.docx [file khvi-14-08-1446719-s001.docx]

**Figure 1S.** Seroconversion rate one month after seasonal influenza vaccination in patients with diabetes mellitus (DM) (bullet) and comparison with non-diabetic subjects (non-DM) (square) if available (reference [diabetes type, age group]).

**Figure 2S.** Seroprotection rate one month after seasonal influenza vaccination in patients with diabetes mellitus (DM) (bullet) and comparison with non-diabetic subjects (non-DM) (square) if available (reference [diabetes type, age group]).

**Appendix 1 – Search strategy**

***PubMed***

*#1. Metabolic disorders or existing comorbidities (i.e. diabetes mellitus type I and II, hypertension, hyperlipidaemia, hypercholesterolemia and atherosclerosis)*

("Metabolic Diseases"[Mesh] OR metabolic disease*[tw] OR metabolic disorder*[tw] OR metabolic syndrome[tw] OR syndrome X[tw] OR MetS[tw] OR "Diabetes Mellitus"[Mesh] OR diabet*[tw] OR NIDDM[tw] OR IDDM[tw] OR T2DM[tw] OR "Insulin Resistance"[Mesh] OR insulin resistan*[tw] OR insulin sensitivit*[tw] OR insulin dependen*[tw] OR "Hypertension"[Mesh] OR hypertensi*[tw] OR high blood pressure*[tw] OR "Hyperlipidemias"[Mesh] OR hyperlip*[tw] OR hypercholestero*[tw] OR hypertriglyceridemi*[tw] OR "Atherosclerosis"[Mesh] OR atherosclero*[tw])

*#2. Influenza*

("Influenza, Human"[Mesh] OR influenza*[tw] OR flu[tw] OR ILI[tw])

*#3. Vaccination*

(“Influenza Vaccines”[Mesh] OR "Influenza Vaccines"[nm] OR vaccin*[tw] OR immuniz*[tw] OR immunis*[tw] OR immunogen*[tw] OR immunit*[tw])

***Cochrane library***

*Metabolic disorders or existing comorbidities*

#1 MeSH descriptor: ‘Metabolic Diseases‘

#2 metabolic disease*:ti,ab OR metabolic disorder*:ti,ab OR metabolic syndrome:ti,ab OR syndrome X: ti,ab OR MetS:ti,ab

#3 MeSH descriptor: 'diabetes mellitus'

#4 diabetes:ti,ab OR diabet*:ti,ab OR NIDDM:ti,ab OR IDDM:ti,ab OR insulin resistan*:ti,ab OR insulin dependen*:ti,ab OR insulin sensitivit*:ti,ab OR T2DM:ti,ab

#5 MeSH descriptor : 'hypertension' OR 'hyperlipidemia' OR 'atherosclerosis'

#6 hypertensi*:ti,ab OR high blood pressure*:ti,ab OR hyperlip*:ti,ab OR hypercholesterolemia:ti,ab OR hypercholesterolaemia:ti,ab OR hypertriglyceridemia:ti,ab OR atherosclero*:ti,ab

**7# (#1 or #2 or #3 or #4 or #5 or #6)**

*Influenza*

#8 influenza:ti,ab OR flu:ti,ab OR ILI:ti,ab

#9 'Influenza, Human'/exp

**10# (#8 or #9)**

*Vaccination*

#11 'influenza vaccine'/exp

#12 vaccin*:ti,ab OR immuniz*:ti,ab OR immunis*:ti,ab OR immunogen*:ti,ab OR immunity*:ti,ab

**#13 (#11 or #12)**

**#14 (#7 and #10 and #13)**

**Embase**

The following search strings were used to search for relevant articles in Embase.

*#1 Metabolic disorders or existing comorbidities*

('metabolic disorder'/exp OR ‘metabolic disease’:ti,ab OR ‘metabolic diseases’:ti,ab OR ‘metabolic disorder’:ti,ab OR ‘metabolic disorders’:ti,ab OR ‘metabolic syndrome’:ti,ab OR ‘syndrome X’:ti,ab OR MetS:ti,ab OR ‘diabetes mellitus’/exp OR diabet*:ti,ab OR NIDDM:ti,ab OR IDDM:ti,ab OR 'insulin resistance':ti,ab OR 'insulin resistances':ti,ab OR 'insulin resistant':ti,ab OR 'insulin resistancy':ti,ab OR ‘insulin dependence’:ti,ab OR ‘insulin dependency’:ti,ab OR ‘insulin dependend’:ti,ab OR ‘insulin dependent’:ti,ab OR ‘insulin dependently’:ti,ab OR ‘insulin sensitivities’:ti,ab OR ‘insulin sensitivity’:ti,ab OR T2DM:ti,ab OR 'hypertension'/exp OR 'hyperlipidemia'/exp OR 'hyperlipoproteinemia'/exp OR 'hypercholesterolemia'/exp OR 'hypertriglyceridemia'/exp OR hypertensi*:ti,ab OR ‘high blood pressure’:ti,ab OR hyperlip*:ti,ab OR hypercholestero*:ti,ab OR hypertriglyceridemi*:ti,ab OR 'atherosclerosis'/exp OR atherosclero*:ti,ab)

*#2 Influenza*

(‘Human influenza’/exp OR influenza*:ti,ab OR flu:ti,ab OR ILI:ti,ab)

*#3 Vaccination*

(‘influenza vaccines’/exp OR vaccin*:ti,ab OR immuniz*:ti,ab OR immunis*:ti,ab OR immunogen*:ti,ab OR immunit*:ti,ab)
